# How do researchers acquire and develop notions of research integrity? A qualitative study among biomedical researchers in Switzerland

**DOI:** 10.1186/s12910-019-0410-x

**Published:** 2019-10-16

**Authors:** Priya Satalkar, David Shaw

**Affiliations:** Institute for Biomedical Ethics, University of Basel, Bernoullistrasse 28, 4056 Basel, Switzerland

**Keywords:** Development of research integrity, Responsible conduct of research, Value education, Integrity education, Inculcation of values, Moral education, In-depth interviews, Switzerland

## Abstract

**Background:**

Structured training in research integrity, research ethics and responsible conduct of research is one strategy to reduce research misconduct and strengthen reliability of and trust in scientific evidence. However, how researchers develop their sense of integrity is not fully understood. We examined the factors and circumstances that shape researchers’ understanding of research integrity.

**Methods:**

This study draws insights from in-depth, semi-structured interviews with 33 researchers in the life sciences and medicine, representing three seniority levels across five research universities in Switzerland.

**Results:**

The results of this study indicate that early education, moral values inculcated by the family and participation in team sports were the earliest influences on notions of honesty, integrity and fairness among researchers. Researchers’ personality traits, including degree of ambition and internal moral compass, were perceived as critical in determining the importance they attributed to conducting research with high ethical standards. Positive and negative experiences in early research life also had a significant impact on their views regarding research integrity. Two thirds of the study participants had not received any formal training in research integrity. Their awareness of training opportunities at their institutions was also limited.

**Conclusion:**

Age-appropriate development of honesty and integrity starts as early as primary education. Research integrity training should be offered from the bachelors level and continue throughout the entire professional life of researchers. Although these courses may not imbue researchers with integrity itself, they are essential to improving the research culture, reinforcing integrity norms, and discouraging researchers who lack personal integrity from engaging in research misconduct.

## Background

Structured training in research integrity (RI), research ethics and responsible conduct of research (RCR) is one of the strategies to reduce research misconduct and strengthen reliability of and trust in scientific evidence [1]. The National Science Foundation (NSF) and the National Institutes of Health (NIH) in the United States require all researchers working on projects funded by them to be trained in RCR [2–4]. Many European universities also recommend training researchers in good scientific practice and research ethics, though the requirements are not uniform across Europe and the curricula and contents of existing courses vary [5–7]. It is believed that RCR training imparted face to face or using web-based platforms could reduce costs incurred by universities in investigating research misconduct, by journals in investigating and retracting fraudulent articles and the resources lost when researchers engage in fraudulent research activities [8]. However, even after two decades of mandated RCR training in the US, the evidence on effectiveness of these trainings in changing behavior of researchers remains inconsistent and weak [2, 9]. Resnik argued that these training programs should not be evaluated in terms of the outcomes of positive behavior change in researchers but rather in relation to their awareness of norms, knowledge of good scientific practice and decision making process [4]. Steneck called for global RCR trainings with standardized course curricula, common objectives and training material to facilitate increasingly collaborative global research and reduce costs of this training [10].

Though the importance of RCR training in shaping researchers’ awareness of good scientific practice is undeniable, its role in changing behavior is less clear. Such training is only one of the many factors that influence researchers’ behavior and scientific practice [11]. Everyone develops individual views on honesty, integrity and fairness, whether or not they are trained as research professionals [12]. In fact, by the time an individual enters a scientific profession, he or she already has certain ideas about what it means to do good and honest work, and what values and principles guide his or her behavior in research activities. Thus, RCR training if available is often imparted to individuals who have already undergone some degree of conscious or subconscious reflection on integrity.

There is limited academic literature exploring the factors that shape researchers’ personal and professional sense of integrity. Though one might refer to someone as a person with a high or strong sense of integrity, what we mean by ‘high integrity’ or how we conclude that someone has a strong or weak sense of integrity often remains unquestioned. It is an assessment we all conduct without explicitly verbalizing the meaning of integrity or teasing out its nuances. Furthermore, it is unclear whether a person’s strong or weak sense of integrity remains static over time or whether it can vary over a spectrum of strength depending on personal and professional circumstances. Qualitative research methods, which are probably better suited to elicit this implicit and understood meaning of integrity, have been used only rarely to investigate the ways in which researchers’ sense of research integrity is instilled and developed. Furthermore, there is limited research on this topic in both the European and Swiss contexts [5].

In order to fill this knowledge gap, we undertook an exploratory qualitative study to understand experiences and perspectives of researchers working in medicine and life sciences in Swiss universities. RCR training is not mandated in Switzerland though some universities have recently started providing such training for their research staff. In this manuscript, we examine factors, circumstances and influencers that shape or mould researchers’ understanding of research integrity and the importance they attach to it. We first describe the ways in which their understanding of research integrity was formed before being exposed to RCR training (if any) in their professional lives. Then we evaluate their views on whether researchers can be trained in research integrity and on strategies to strengthen standards of integrity in spite of academic career pressures. Finally, we elaborate on their experience (if any) with research integrity training and their awareness of possibilities to be trained in research integrity at their current academic institution.

## Methods

### Aim

Our aim is to examine the factors, circumstances and influences that shape or mould researchers’ understanding of research integrity. We elaborate on how the concept of research integrity evolves over lifetime of researchers in response to their personal circumstances and work environment.

### Study design

This manuscript is part of an exploratory qualitative research study called PRISM (Perspectives on Research Integrity in Science and Medicine) which was aimed at understanding experiences of researchers in Switzerland regarding research integrity related topics through in-depth qualitative interviews. This study was conducted from 2016 to 2018 and explored various topics such as authorship practices, disclosure of conflicts of interests, experience of witnessing scientific misconduct, raising concerns about misconduct and knowledge and awareness of institutional, national and international guidelines on research integrity [13–15].

### Sample and setting

This study draws insights from 33 researchers in life sciences and medicine across five research universities in Switzerland. Two of these are in the French speaking and three in the German speaking regions of the country. We included researchers at three seniority levels. Five junior researchers were MD or PhD students, 17 mid-level researchers represented postdoctoral fellows, group leaders and chief physicians, and 11 professors constituted the category of senior researchers. Eleven of 33 respondents were women; their distribution across three seniority levels reflects the same gender ratio as in research work force in these three categories across Swiss academia. Eight respondents worked exclusively in laboratory settings whereas the others were engaged in clinical and/or translational research.

### Data collection

Researchers in the life sciences and medicine were approached through the mailing lists of concerned departments and professional networks of the Swiss Clinical Trial Organization (SCTO) and Life Sciences Switzerland (LS2). We prepared a flyer describing the study objectives and the ways in which respondents’ identity would be protected throughout the research. They were asked to contact us if they were interested to be part of the study. PS made appointments for individual interviews at the time, date and location convenient for the respondent, in person or over the phone or in a skype call. We are aware that in-person interviews often allow better rapport building than a phone conversation. However, we firmly believed that respondents must be able to choose the means that they found most suitable especially because they were going to narrate some highly sensitive experiences related to research integrity. Ten respondents chose to be interviewed in person whereas the others were interviewed either through a phone or a skype call. PS conducted all interviews in English between May and December 2016. At the beginning of each interview, she described the goal of the study, the procedure of data anonymization and analysis, and clarified any questions respondents might have about the study. She obtained oral informed consent and permission to record the interview on an audio device before starting the interview. Oral informed consent was also recorded on the audio-device. The interviews were guided by a semi-structured interview guide. We prepared this interview guide drawing insights from relevant literature, obtained feedback from project collaborators, and pilot tested the guide with two respondents, one from life sciences and the other from medicine. After careful consideration, the research team decided to include pilot interviews in the final data set. The interview duration ranged from 28 to 110 min, with the average being 65 min. The interest of respondents in the topic, their experiences and other scheduled appointments often influenced the duration of the interview. To accommodate the busy schedules of some of the senior researchers, we interviewed them over multiple meetings or phone calls of shorter duration. PS led the verbatim transcription of interviews together with two assistants who had signed confidentiality agreements. We removed all personal identifiers such as the name of the respondent, university and department where they worked, their prior university affiliations and any other researchers or institution they mentioned during the interview. PS verified each transcript against the recording and made corrections. In a few cases, where we needed to obtain further clarification on a point under discussion, we approached the respondent again and asked additional questions either through a phone call or in an email. We had offered respondents possibility to read the transcript but no one opted to do so. Each transcript was saved with a consecutive respondent number (R1, R2, R3 and so on). PS maintained a master list with respondent identity and their assigned number. We stopped recruitment when we were convinced that additional interviews were not providing any new insights regarding our research objective.

### Data analysis

Both researchers read the transcripts several times to familiarize themselves with the data. PS undertook deductive coding using qualitative data analysis software MAXQDA (licensed by the university) and DS carried out manual coding. DS and PS discussed codes extensively and any differences were resolved through discussion. Following procedures of thematic analysis codes were built into themes [16]. In this manuscript, we focus exclusively on ways in which respondents believed their sense of research integrity developed and the role research integrity training played in their scientific work. We did not analyze the data at the level of different universities or linguistic regions of the country for two main reasons. First, the sample of this exploratory qualitative study is rather small. If data were analyzed at the individual university level, the numbers of researchers representing particular universities would have been even smaller. Furthermore, the views from such a small number of participants do not represent the prevalent practices relating to research integrity of entire university, especially if we also take into account the self-selection bias inherent to our methodology. This would have posed particular problems also during publishing the results of this study. The universities whose researchers actively participated in our study could be seen in a negative light due to experiences shared by a few researchers whereas in spite of prevalent research integrity related issues, a university could be seen in a positive light simply because not enough researchers from that university took part in this study. The second reason for analyzing the data at aggregate national level was to ensure participants’ confidentiality. The professional community in medicine and the life sciences in Switzerland is close-knit and has number of established inter-university and interdisciplinary collaborations. Therefore, with the gender, the seniority level and the affiliation of a particular respondent, one could in principle guess or even correctly identify a respondent. Such clues towards the identity of respondents could very probably harm their career.

## Results

Our aim was to examine the ways in which researchers’ notion of research integrity (RI) is developed and shaped. In order to elaborate on our results, we will focus on three main themes and their corresponding subthemes as described in the Table 1.

**Table 1:** Themes and subthemes

Themes	Subthemes
Development of sense of integrity	Early education and upbringing
	Influence of researcher’s personality traits
	Influence of external factors, experiences and role models
Can researchers be taught research integrity?	Yes it can be and should be taught
	It can be taught but only to an extent
	You either have it or you don’t
Role of research integrity training in shaping or strengthening integrity	Received formal RI training at any point in career
	Awareness of training opportunities in current institution

### Development of sense of integrity

All respondents explained that this was their first attempt to set out the origins of or influences on development of their own understanding of research integrity, though many had thought about it in other situations related to their personal and professional life. We categorized their reflections into three subthemes: early education and upbringing, attributions to personality traits and influence of external factors, personal experiences and role models.

#### Early education and upbringing

Fourteen respondents explained that their sense of integrity was strongly influenced by their early education and upbringing, specifically the values their parents and teachers had inculcated in them. They traced the origins of their professional and research integrity to the values of honesty, responsibility and trustworthiness together with moral lessons taught by their parents such as ‘do not lie’, ‘be fair’, and ‘do not cheat’. They argued that individuals already have their ideals or behavioral limits by the time they start their research careers. Therefore, informal education in integrity has to start very early in one’s life even before one makes a conscious decision to follow a career in science or research. Two respondents (R9 and R31) specifically mentioned religious education during childhood as having significantly influenced their sense of integrity. Two others (R1 and R15) remembered that they were not able to enjoy their victory in sports when it was achieved through cheating. They felt uneasy and unhappy about their inappropriate actions or behavior. One respondent (R28) believed that his attitude of being honest, correct and helpful was a general attitude rather than a trained one, acquired through a continuous process but also added that his upbringing ‘did not harm’ the process.

I think it was the early childhood education, the values that your parents live with, they transfer it to you where they make clear that these values are important for a social behavior. And I think sports is the other point, team sports, to learn to play fair. **R15, male, senior researcher**

A big part of it is from my family. My mother instilled in me at a very early age a very strong work ethic….. Honesty was something that was just expected, that was something very important, so really from a very young age, I absorbed it. **R33, female, mid-level researcher**

One respondent shared her personal anguish when her child lied to her for the first time. Her response in that situation was critiqued by the others to be out of proportion; however she remained firm that this behavior must be corrected at the earliest.

….It is true that when my child lied for the first time when he was young, I was completely disturbed, completely. …. I made a big thing out of it which I still think was right because then he never did it again. But other people told me that you are making it a big thing and it is a child and it is normal but I still felt like ya, but if you start like this, you never know… if you want me to be able to trust you, then I should be able to trust you. Then I give you more freedom and that is the basis of our relationship. **R27, female, mid-level researcher**

#### Influence of researcher’s personality traits or character

Eleven respondents said that their innate personality traits or character had influenced their notion of research integrity. Many of these stated that they had a strong sense of justice from an early age, which became evident in their relationship with their parents and siblings. A few referred to their desire to be able to sleep well at the end of the day, which they thought would be impossible if they were cutting corners or were cheating in their research. In their professional lives, they often looked for people with similar values to work with; they stopped collaborating with colleagues who did not value these qualities the same way as they did.

I think it is because I just do not like lying. If I do something wrong, I feel bad about it. If I would cheat in the lab, I would have enormously bad conscience and it will show. I cannot hide it. …. and over years this only grew stronger or maybe I became more aware of this myself. **R22, female, mid-level researcher**

When you start your own research, it is just a gut feeling somehow. You read all about how it (research) should be done and you do it differently, then you do not feel very well because you know this is not how you should do it. … I feel my guts are saying: I do not feel really well. Even when I go home, I think I should not do it like this because once it is published, people can come back and say- are you sure this is how you should do it? And then they can question your whole career or the things you have published. **R25, male, junior researcher**

This particular junior researcher was quite shocked when his supervisor told him to do things which, he had learned through taking courses on methodology, were not in line with good scientific practice. He wondered whether confidence in deviating from standard practice without feeling bad about it grows in line with seniority.

One of our senior respondents discussed in details how he learned the ‘rules of the game’ for academic success and learned to play it well. However, his internal critic compelled him to re-evaluate his approach to science and definition of success.

I also got addicted I think a little bit to the fact that you can play this game and if you play it well, you are successful to some degree….. but maybe in my heart (laughs) or in my soul or whatever you want to call it. I knew this is not the way it should be… This will not improve the human condition which is basically at the end of the day what we are trying to do. **R11, male, senior researcher**

Two researchers specifically discussed the influence of the ambition, career goals and personal criteria for success that researchers set for themselves on their attitudes towards research integrity.

Ethics also depends on how hungry you are about your career and how you balance ethics and career. **R4, male, mid-level researchers**

Participants reflected on the relationship between gender, ambition and societal expectations. Male researchers often felt stronger societal and familial expectations that they would succeed and obtain permanent academic positions whereas, it was fine for women researchers not to aspire for or reach similar positions due to their role in raising families. One of our female respondents elaborated on this perceived difference. In the German-speaking world, ‘habilitation’ is an additional research qualification required from researchers to be considered for a permanent academic position. It requires a researcher to establish research expertise in a particular field other than their doctoral research, proven by a track record of high impact, peer reviewed publications in addition to a teaching qualification. These titles are highly valued in academia in the German speaking part of Switzerland. However, for this particular respondent, the title of habilitation was not a personal yardstick of success. Therefore, in spite of having fulfilled all the requirements for obtaining habilitation, she did not rush through the process to obtain habilitation and continued working in her field.

May be one thing that I do not like so much and may be that is also something typical for women…….. I think, for me it is not the title that counts but it is your work. I took quite a long time to do my habilitation and my boss was mad about it. Here we need 10 or 15 papers and I had 35 when I did it. But it was not important for me. I mean the work was important but university considers that they have people doing good things only when they have titles. **R8, female, mid-level researcher**

Three respondents (R6, R8 and R23) described that they self-initiated learning, reading on the topic and thinking about research integrity. In the absence of formal training, reading and reflection was their way to strengthen research integrity. This was often triggered by challenging situations they experienced in their work environment, which left them feeling uneasy and restless. One junior researcher who had raised concerns about misconduct of her supervisor turned to online resources, discussion forums and reading to learn more about research integrity.

… a lot of my ethics and research integrity comes from reading these different experiences online because otherwise, if I was just shaped by my supervisor, I would have gone in the same direction as him. **R23, female, junior researcher**

#### Influence of external factors, experiences and role models

Twelve respondents described the influence of research environment, earlier experiences in research career and role models and supervisors on their sense of research integrity. Two respondents (R2 and R12) believed that, although intrinsic values of honesty and integrity should come naturally from within and stay strong irrespective of external circumstances; the research environment with all its pressures can push researchers towards compromising their integrity. Sometimes, financial freedom and stability in terms of conducting one’s own research or leading a research group, allows researchers to work with the high standards of integrity they aspired for in their life. However, if they are dependent on some other researchers or principal investigators (PIs) whose values regarding research integrity may not match those of researchers, conflicts occur. While reflecting on his disappointment about a prestigious research institute abroad in terms of the lack of respect given to research integrity, Respondent R11 described how his eventual success in obtaining a large research grant and working as a principal investigator allowed him to follow the high standards of integrity which he had always aspired to.

….. (while abroad), I wanted to get on with my career so I played along to a certain degree (by the rules of the group leader) and then I came back to Switzerland. I am a PI now; I have my own budget, my own employees, my own project. Nobody tells me what to do and how to do it. I was very lucky to receive good funding….. I think I would be kidding myself if I did not take this fact into equation. The fact that I am no longer under this big pressure, this of course helps your scientific integrity a lot. I try to be as scientific as I can and I really think about this on a daily basis. **R11, male, senior researcher**

This respondent shared his one sentence definition of research integrity influenced by a famous quote by Charles Darwin he had once read. ‘A scientific man ought to have no wishes, no affections, - a mere heart of stone’. This quote reminds him of the importance of objectivity in his research work, in spite of all the external temptations, pressures, personal ambitions or goals. He aspires to have a heart of stone because considering his hopes and dreams could influence and bias the way he interprets his data.

Respondents often reflected on positive as well as negative career experiences that shaped their values regarding research integrity. The most positive experiences came through opportunities to work with senior researchers with strong research integrity who became mentors and role models to junior researchers. Seven respondents (R4, R6, R8, R12, R16, R17, R18) learned through observing their mentors who did not just preach about integrity but practiced it in spite of all the pressure and were still successful in terms of the valuable work they carried out. In contrast, two respondents (R17 and R23) also described the strong impact the negative professional experiences had in strengthening their research integrity.

I worked in a lab where one of the clinicians and the boss of the study did some fraud, they had to retract one of their papers and this also costed my boss his job at that institution. It was a strong lesson and it kind of scared me. May be that is also some way that you show, what happened to people that have done fraud. It is kind of negative picture but in medicine that will probably work. **R17, male, senior researcher**

… it was from experience, experiencing others, their pressure to publish or perish.. and seeing people that I knew were very good researchers with high ethics and high efforts, they do not get grants because they do not have enough publications and knowing people that I knew do not do things right but have 20 publications, so they get the grant. Though this was not directly affecting me, I saw it happening over and over again… so that is when I seriously started reading about this (research integrity). **R23, female, mid-level researcher**

### Can researchers be taught research integrity?

Respondents preferred the term ‘sensitization’ via research integrity courses over the word ‘training’. They argued that some degree of intrinsic or early childhood values of being honest and fair are a prerequisite to strengthening research integrity, especially in a context of compelling external factors and pressures that push researchers towards compromising honesty and integrity in their work. Reflections on this topic were provided by 27 out of 33 respondents. We categorized them into three subthemes.

#### Yes: it can be taught and should be taught

Thirteen out of 27 respondents argued strongly that research integrity can be and should be taught like any other skill. They all argued for including components of RI training from the undergraduate courses, building on it throughout the professional career and also conducting refresher trainings at regular intervals.

I think there are a lot of people who have a good heart and they mean good... the circumstances turn them bad and then there is kind of like having a devil on your left shoulder and an angel on your right shoulder for instance and then they talk to each other and it's a fight and someone wins...and maybe we can strengthen the angel a little bit, by giving them examples and telling them, actually what happens when you don't adhere to it (research integrity) and what can happen...what beautiful things can happen when you do adhere to it... **R11, male, senior researcher**

It is 100% training. I mean it **has** (emphasis in original) to be. Of course, we all carry on our own virtues and ideas about the world but this is something that needs to be taught and people need to have proper training. You cannot expect every individual out there who experiences these challenges and pressures to put morality and ethics as no. 1 in their agenda. So this is something that needs to be integrated into formal training of future scientists. **R32, male, mid-level researcher**

#### It can be taught but only to an extent

One third of respondents (nine out of 27) believed that research integrity has components that could be transferred during teaching or training. However, they also stated that to acquire such knowledge and apply it in one’s work, one must have at least some intrinsic value of honesty, integrity and openness to learn, together with a strong willpower and commitment to put these values into practice, even if working with high levels of integrity could reduce or delay career opportunities and growth. They argued that there are limitations to the extent to which training in research integrity can be effective, but they did not rule out the importance and responsibility of training researchers in research integrity related topics. Their ambivalence regarding the limits and utility of RI training is expressed in the quotations below.

…let’s say that research integrity, you can formally talk about what is scientific misconduct, what is plagiarism; you can formally talk about it. But it is, I don’t know the English word for it, it is ‘eine innere haltung’ [an inner attitude] not to do it. **R13, male, senior researcher**

I think people with right attitude happen to be more open to integrity questions. I don’t think that you can train attitudes. Training is possible if one is willing to at least overcome false ideas or whatever was just not known. But if somebody has certain attitudes towards publishing or career ideas, I don’t think training would have an effect. **R28, male, mid-level researcher**

I think it is a bit of both. Personal integrity is very closely linked to the way you conduct yourself in the lab. But training and speaking to people about the subtleties that exist, could go a long way. …. if you are the type of person that is going to completely fabricate data, no amount of training could fix it or could influence it. But especially for the things that maybe people don’t realize could constitute misconduct, training could go a long way at least to make people aware of it. Make it part of the conversation that it can become something to consider when you are planning your experiments. **R33, female, mid-level researcher**

#### You either have it or you don’t

Finally, five out of 27 respondents argued that research integrity is an intrinsic value and a researcher either has it or s/he doesn’t. They were not convinced that one could be trained in research integrity.

I think that it actually boils down to everyone’s own feeling of integrity and this is probably very hard to teach. What can be done is to have reminders about research integrity, [….] maybe that will add a layer of a brake if they ever want to breach their integrity. But teaching itself; I don’t think… there is always a side that wants the easy way. **R1, male, mid-level researcher**

### Role of research integrity training in shaping or moulding research integrity

Respondents stated that formal RI training had limited influence on shaping their notion of research integrity. They also demonstrated little awareness of formal training opportunities in RI at their own research institutions.

#### Received formal training in RI at any point in time during career

20 out of 33 respondents in our study said that they had not received any formal training in research integrity at any stage in their career. Those who received formal training most probably had those opportunities during their studies or fellowships in North America or through their association with the pharmaceutical industry. When asked to describe the contents of training, eight out of the 13 respondents referred to training in good clinical practice (GCP) or good laboratory practice (GLP), neither of which is actually a research integrity guideline. One respondent described having completed online RI training out of personal interest. The remaining four had a lecture or two on research integrity related topics at some point in their career.

Respondents were divided in their opinion regarding the utility of the formal RI training they received during their research/studies in North America. One respondent was strongly in favor of the mandatory, modular, online training in research integrity required in many universities in the US. Another respondent reflected positively on his experience with RI training during his master’s studies at a Canadian university. He reflected on differences in research culture and focus put on research integrity training in the Canadian versus in Swiss/German contexts.

… when I was a master’s student, it (RI training) was propagated as a very **fundamental** (emphasis in original) concept. So every student had to take this intensive course in research integrity to start their studies otherwise no one could start research. It was brought up in different contexts throughout my studies there. Here, not everyone gives research integrity a stage. I have even heard the former deans saying, ‘ohh we do not have a problem with research integrity’. … Every university has issues with that and there need to be rules in place and there need to be knowledge transfer in place so that all researchers actually know about research integrity. **R16, male, mid-level researcher**

However, we also had one respondent who was critical of the North American model of research integrity training.

In the US, I have to say, we had research integrity training, we had sexual harassment training, we had all kinds of trainings. To me, they often served only to a limited extent their purpose and were often given, that was my impression, just to satisfy the bureaucrats. So something that they could put aside and say that everybody got trained once a year and if something happens, then it is not our fault. That should not be the spirit or philosophy behind these courses. It should really permeate the education and should be ingrained in people. **R26, male, senior researcher**

#### Awareness of RI training opportunities in current institution

We asked respondents to describe training opportunities in research integrity available at their current institutions. A total of 22 out of 33 respondents said that they did not know of any such training possibilities. One of these respondents strongly argued that if such trainings are available at an institution, they should be clearly visible and ‘in the face of researchers’; one should not have to search for those in course catalogues. Another respondent believed that there might be some form of training under transferable skills courses or continued education programs. Ten respondents named a few courses available at their institutions on research integrity related topics. Four of these were themselves actively involved in conducting GCP and GLP trainings and they mainly referred to those courses. The most commonly mentioned course was on preventing plagiarism.

## Discussions

The results of this study indicate that early education, moral values inculcated by the family, and participation in team sports were the earliest influences on notions of honesty, integrity and fairness among researchers. Researchers’ personality traits, including degree of ambition and internal moral compass, were perceived as critical in determining the importance they give to conducting research with high ethical standards. Experiences in early research life, both positive and negative, also had significant impact on their views on research integrity. A large majority of respondents believed that researchers can be trained in research integrity to some degree but in order to receive such training, to be willing to reflect on one’s behavior and to put this knowledge into practice, the researcher would need a certain degree of openness towards this topic and interest in learning. For some researchers with a weaker sense of integrity, this attitude might be lacking, making training ineffective. Exposure to RCR or RI trainings during professional life can only be built on the moral values researchers already have. Respondents argued that RCR training will not prevent those who do not value integrity from engaging in research misconduct but will definitely help create awareness about ethical scientific practice among researchers. Finally, a relatively small number of researchers who participated in this study had received formal training in RCR or RI, their awareness of training opportunities available at their research institutions was rather limited and they shared ambivalent views on the effectiveness of the RI/RCR training they received on shaping their behavior. A majority of those who believed they had taken courses in RI or who were involved in providing RI training themselves referred to the courses in GCP and GLP as courses in RI.

### Catch them early, catch them young

Early childhood education and upbringing was described by participants as the key influence on their attitude towards research integrity. Parents, school teachers and sports coaches were the first influences on integrity, reinforcing the importance of honesty in all aspects of life and discouraging cheating as a means to get ahead of others in competition. Currently, RCR or RI training is normally provided during masters or doctoral programs, wherever it is provided. By the time a person enters these professional training programs, they often have some personal notion of correct behavior in the research context, what the limits or boundaries of their values are in case they find themselves in an ethically challenging situation and whether they would be willing to compromise research integrity in order to succeed or to fit into the culture of a research group. In other words, while it is possible to teach researchers at this stage research integrity rules, it might be far too late to imbue them with integrity that they do no already have.

We believe that multiple opportunities in early lives of individuals to reinforce and strengthen integrity should not be missed. Moral (and religious) education, school education and sports training together with parental upbringing could harness and inculcate higher importance to values of honesty and integrity and later adherence to these values in challenging situations in personal and professional life. The 2009 Guidance document from the NIH on teaching responsible conduct of research in academic settings recommends that the students need to be exposed to principles and relevant elements of research integrity to their level of understanding and scope of their studies as early as during bachelors program, repeated periodically throughout their training as research professionals and even when one receives academic positions [17]. Researchers are exposed to different levels of stress and external pressures along their professional trajectory and periodic discussion on research integrity could work as necessary boosters or stimuli for researchers to reassess their values, to reflect on their work and to correct their behavior if needed [18]. Going further, the importance of academic honesty and fair play should also be stressed in earlier academic settings such as primary and secondary school, as teachers can be important role models for children whose sense of integrity is still developing. An increasing emphasis on critical thinking and objectivity in schools will hopefully also encourage stronger personal integrity. One might argue that teaching integrity at primary and secondary schools is unrealistic due to the number of schools involved. However, we believe that the effort to strengthen young student’s values of honesty and integrity would be a foundation for personal and professional integrity in any field they might chose to work in later life. We do not foresee special courses in integrity for these schoolchildren, rather that these values could be discussed in various settings and other classes that students have to take anyway, such as critical thinking or personal development classes. Resnik and Stuart have argued in favor of expanding RCR training to all students, trainees, faculty and staff involved in research [18]. While we support this, we think that greater emphasis in the development of honesty, integrity and fair play in an age appropriate manner as early as in primary and secondary school could result in students who are already highly sensitized to integrity entering professional courses and research training programs.

### Hiring researchers

In current academic settings, researchers are often hired on the basis of their qualifications, academic grades and scientific output in terms of publications. Though these parameters can be indicative of scientific excellence, they do not take into account the core values researchers bring with them and their willingness to compromise integrity in order to succeed in their careers. Assessing these values during the hiring process may not be easy; however, some discussion with the candidates to understand their internal value system, factors that drive them in face of failure, setbacks and extreme competition could give valuable clues towards personality traits and character. Ambition by itself is not a negative trait but when uncontrolled it could lead to researchers engaging in a series of questionable research practices and even scientific misconduct in order to succeed especially if the external checks and balances to prevent such behavior are weak or absent [19].

We are aware that such screening to assess persons’ internal values may not identify all researchers with weak sense of integrity. Individuals with weak integrity could be smart enough to project ‘fake’ highly ethical values during the hiring process in order to get jobs. However, focusing on values could send a strong message to all researchers that research integrity, high ethical standards, accountability, responsibility and fairness are just as important as publication record and academic grades. Irrespective of the position for which a person is hired, robust supervision and mentorship in the early months of person’s integration into a new team will be the next opportunity to assess whether the impression given during the hiring process with regards to values is also translated into day to day work.

### Role models and mentors

A large number of respondents in this study discussed the importance of positive and negative professional experiences and role models in their lives. For a few of them, negative role models were the biggest influence in order to learn more about research integrity and responsible conduct of research. A few others identified the consequences of research misconduct on the lives of researchers in question, their research team and collaborators when caught as one of the strong influences compelling them to review their own behavior and research practice. It should be noted that some researchers might be abiding by good research practices not out of a deep sense of integrity alone, but because they are scared of the potential career consequences of being caught; in contrast, to other researchers with high integrity who would not need to consider the consequences, but would simply do what is right. Another paper related to this project explores this distinction in more depth [13]. While the outcomes might be the same whether the motivation is fear of being caught or a deep sense of integrity, having good research behaviors be motivated by internal rather than external factors (or both) seems likely to be more reliable. The remaining researchers credited their mentors and supervisors, who not only talked about research integrity but also practiced it even in extremely challenging circumstances as the strongest influence on their professional life. Such examples made them aware that one can have successful research career and meaningful scientific contribution while working with the highest standard of ethics and integrity.

Some of the participants of this study stated that discussing cases of scientific misconduct and their consequences on researchers, discipline and society at large could be a good strategy to deter researchers from engaging in research fraud and misconduct. Drawing on the research assessing the impact of publishing health risk warnings with vivid pictures of patients with cancers on the cigarette packaging on cigarette purchasing behavior of smokers or attempts at cessation [20], we argue that simply highlighting ‘fallen cases’ of scientists due to misconduct may have limited influence on shaping research integrity of research professionals. Not all cases of scientific misconduct become publically known [21]. Some might believe that those who got caught in scientific fraud were not smart enough to get away with the fraud and that they themselves are better at covering the tracks of their fraudulent research practices in order to avoid being caught. Indeed, teaching about “fallen cases” might help teach unethical researchers how to avoid detection. We agree that positive role models of researchers with high sense of research integrity will be more effective in shaping ethical research practices [11]; as Wolpe puts it, “All the formal ethics training in the world cannot compensate for an unethical mentor.” [22] Similarly, an academic system that values and rewards researchers who work with high standards of integrity will provide more positive reinforcement than only focusing on the impact of proven misconduct on the lives of those engaged in misconducts and their collaborators [19].

### Significance of structured RI or RCR trainings at institutional levels

The participants in this study had little experience with RCR or RI training and those who had often referred to having taken or taught courses in GCP and GLP. They also had limited knowledge about training opportunities in this area within their university. In spite of the inconsistent evidence regarding the effectiveness of RI and RCR training in shaping research behavior and practices, we argue that such training courses must be offered by universities and that research staff should be made aware of their availability. These trainings will be valuable to a large majority of researchers who do believe in the importance of conducting research with high ethical standards but who could be unaware of all the guidelines, norms and policies around research integrity. Those exposed to RI or RCR training are also likely to remember these norms when confronted with morally challenging situations in their professional lives.

If someone believes that research integrity is not important for them in their work, no amount of RCR training is going to change that view. However, rigorous supervision, internal peer review by research colleagues and external checks through institutional policies and structure could have some control on behavior of such researchers and what they are allowed to get away with [23]. Training researchers in the basics of research integrity and raising awareness of integrity rules also encourages them to raise concerns about potential misconduct; if no training is offered, it sends the message that researchers should not make waves by whistleblowing.

RCR training will strengthen faith in research integrity among those who value honesty, transparency and trustworthiness in their work. It will also have some influence on those who are undecided and are at risk of compromising research integrity if the external pressure to succeed gets too high. As one of the respondents in our study stated, RCR training for such individuals will be one of the ways to strengthen ‘the angel’ sitting on one shoulder whereas institutional structures, rigorous supervision and internal peer review will keep ‘the devil’ sitting on the other shoulder in check.

Many researchers know what is right but lack the willpower to do what is right; in this sense they suffer from *akrasia*, or weak-willedness. Their willpower will be improved if training is imparted by those who demonstrate and practice strong commitment to research integrity. However RCR or RI training will have limited influence in shaping researchers views and practices if they are conducted only to fulfill the requirements mandated by institutions or funding agencies.

#### Strength and limitations

The strength of this research lies in its qualitative methodology, which helped uncover the implicit meaning that researchers attach to research integrity and way these values are shaped in an individual’s life. To our knowledge, this is the first study to explore in depth how researchers’ sense of integrity developed across their lives. Furthermore, this is also first attempt to understand experience of researchers across three seniority levels in life sciences and medicine in Swiss academic context.

However, the study also suffers a number of weaknesses attributed to its methodology. First and the foremost, the results of this study cannot be generalized although they provide valuable insight into knowledge, awareness and thought process of researchers in Switzerland regarding research integrity. Respondents of this study self-selected themselves as elaborated in the methodology and hence the results of the study need to be interpreted keeping self-selection bias in mind. Those who were particularly interested in the topic of research integrity or who had challenging and negative experiences regarding research integrity in their professional career are more likely to have responded to our call for research participation. Thus the views of these researchers might be different than a large majority of Swiss research work force who either was not interested in the topic or who had not experienced research integrity related issues in their work environment. In spite of our attempts to include researchers across three seniority levels, the participation by junior researchers was minimal. We believe that junior researchers had rather limited awareness or experience of research integrity challenges and hence participating in this study was not their priority. Finally, we did not have uniform representation of researchers from all Swiss universities.

## Conclusion

Though qualitative in nature, our study suggests that researchers who have high integrity tend to acquire their strong values as children, and that researchers with weak integrity are unlikely to acquire it as adults. Therefore, one of the ways to reduce research misconduct is to increase efforts to nurture personal integrity in schools, while also enhancing and extending research integrity training at the university level. Although these courses may not imbue researchers with integrity itself, they are essential to improving the research culture, reinforcing integrity norms, and discouraging researchers who lack personal integrity from engaging in research misconduct.

## Data Availability

Our transcripts and analysis files are not publically available. We do not have permission from our respondents to make their interview data set public as it contains sensitive information about their experiences with breaches of research integrity and scientific misconduct.

## Abbreviations

GCP: Good Clinical Practice
GLP: Good Laboratory Practice
PI: Principal Investigator
RCR: Responsible Conduct of Research
RI: Research Integrity
